# Cranberry Pomace Extract Exerts Antiviral Activity against Zika and Dengue Virus at Safe Doses for Adult Zebrafish

**DOI:** 10.3390/v14051101

**Published:** 2022-05-20

**Authors:** Laura Tamkutė, Juliano G. Haddad, Nicolas Diotel, Philippe Desprès, Petras Rimantas Venskutonis, Chaker El Kalamouni

**Affiliations:** 1 Unité Mixte Processus Infectieux En Milieu Insulaire Tropical, Université De La Réunion, INSERM U1187, CNRS UMR 9192, IRD UMR 249, Plateforme Technologique CYROI, 94791 Sainte Clotilde, France; laura.tamkute@ktu.lt (L.T.); juliano.haddad@univ-reunion.fr (J.G.H.); philippe.despres@univ-reunion.fr (P.D.); 2 Department of Food Science and Technology, Kaunas University of Technology, Radvilenu, pl. 19, LT-50254 Kaunas, Lithuania; rimas.venskutonis@ktu.lt; 3 Diabète Athérothrombose Thérapies Réunion Océan Indien (DéTROI), Université de La Réunion, INSERM, UMR 1188, 97490 Saint-Denis de La Réunion, France; nicolas.diotel@univ-reunion.fr

**Keywords:** zika virus, dengue virus, phytocompound, antiviral activity, agri-food byproduct, cranberry, zebrafish

## Abstract

Mosquito-borne dengue virus (DENV) and zika virus (ZIKV) infections constitute a global health emergency. Antivirals directly targeting the virus infectious cycle are still needed to prevent dengue hemorrhagic fever and congenital zika syndrome. In the present study, we demonstrated that Cranberry Pomace (CP) extract, a polyphenol-rich agrifood byproduct recovered following cranberry juice extraction, blocks DENV and ZIKV infection in human Huh7.5 and A549 cell lines, respectively, in non-cytotoxic concentrations. Our virological assays identified CP extract as a potential inhibitor of virus entry into the host-cell by acting directly on viral particles, thus preventing their attachment to the cell surface. At effective antiviral doses, CP extract proved safe and tolerable in a zebrafish model. In conclusion, polyphenol-rich agrifood byproducts such as berry extracts are a promising source of safe and naturally derived nutraceutical antivirals that target medically important pathogens.

## 1. Introduction

Mosquito-borne dengue virus (DENV) and zika virus (ZIKV) are enveloped RNA viruses belonging to the flavivirus genus of the *Flaviviridae* family [1,2] Flavivirus-associated diseases are a global health emergency [3] infecting at least 400 million people annually via the four serotypes of DENV (DENV-1 to DENV-4). Half a million are diagnosed with the life threatening illness severe dengue (or dengue hemorrhagic fever) [2]. The recent emergence of highly pathogenic ZIKV strains in South America have been associated with unprecedented severe complications in humans grouped together under the umbrella term Congenital Zika Syndrome (CZS) which includes microcephaly in newborns [4,5,6,7,8]. ZIKV can be detected in urine and semen for prolonged periods after infection [9,10,11,12].

The flavivirus genome consists of a single-stranded positive-sense RNA of around 10.7 kb that encodes a large polyprotein that is processed into three structural (C, prM and E) and seven nonstructural proteins (NS1, NS2A, NS2B, NS3, NS4A, NS4B and NS5) [13]. The non-structural proteins are involved in viral RNA replication, morphogenesis and evasion from host immunity. The early stage of virus entry into the host cells is mediated by the E protein [13]. Viral attachment to the host cell surface is mediated by the interaction between the phosphatidylserine exposed on the surface of the viral envelope and the cellular receptor Axl, which belongs to a family of tyrosine kinase receptors. Following AXL-mediated attachment, the virus particle moves across until it encounters a pre-existing clathrin-coated pit. It is then internalized in late endosomes where low pH-dependent membrane fusion between the viral envelope and host vesicles leads to viral genomic RNA release in the cytosol [14]. Viral RNA replication and assembly of viral particles occur in the environment of the ER membranes [15]. The newly synthesized immature particles are transported through the Golgi complex to the extracellular environment [15].

The development of efficient natural therapeutics against DENV and ZIKV infection has become a priority because of the risk of CZS, especially in regions infested by mosquito vectors of disease [8,16,17,18]. It has been reported that plant extracts rich in flavonoids, terpenoids, coumarins, polyphenols and even some essential oils may inhibit DENV and ZIKV infection [19,20,21,22,23,24,25,26,27,28]. Previous studies showed that epigallocatechin gallate (EGCG), delphinidin, isoquercitrin, resveratrol, geraniin and curcumin inhibit ZIKV and DENV infection at different stages of the viral cycle [20,23,29,30,31,32,33]. Antiviral activity also comes from the extracts of small, pulpy berries, a common food source [34,35,36,37,38]; therefore we evaluated whether a polyphenol-rich cranberry byproduct recovered after cranberry juice pressing could prevent ZIKV and DENV infection in vitro.

## 2. Materials and Methods

### 2.1. Cells, Viruses and Reagents

Human lung epithelial A549 cells (ATCC, CCL-185, Manassas, VA, USA), Vero E6 cells (ATCC, ref. CRL-1586) and human-derived Huh-7.5 hepatoma cells (ATCC, PTA-8561) were cultured in a minimum essential medium (MEM: Gibco/Invitrogen, Carlsbad, CA, USA) supplemented with 10 or 5% of heat-inactivated fetal bovine serum (FBS), 2 mmol/L L-Glutamine, 1 mmol/L sodium pyruvate, 100 U/mL of penicillin, 0.1 mg/mL of streptomycin and 0.5 µg/mL of fungizone (PAN Biotech, Aidenbach, Germany) under a 5% CO_2_ atmosphere at 37 °C. The clinical isolate PF-25013-18 of ZIKV (ZIKV-PF13) has been previously described [39] as has the recombinant molecular clone of ZIKV-MR766 that expresses the autofluorescence reporter protein GFP (ZIKV^GFP^) [40]. The clinical isolates of DENV-2 (strain UVE/DENV-2/2018/RE/47099, passage history P4) from Reunion Island in 2018 and DENV-4 (strain UVE/DENV-4/2012/GF/CNR_16008, passage history P3) from French Guyana in 2012 were provided as lyophilizates by the H2020 Project “European Virus Archive goes global” (EVAg). Before DENV production, lyophilizates were resuspended in 200 μL of distilled water. All viruses were subsequently amplified in Vero E6 cells. DENV and ZIKV titration was carried out by plaque-forming assay on Vero cells as previously described and expressed in PFU/mL [22]. Epigallocatechin gallate (EGCG) was purchased from Sigma-Aldrich. Anti-pan flavivirus E monoclonal antibody 4G2-Alexa Fluor 594 was purchased from RD Biotech (RD-Biotech, Besançon, France). A culture medium supplemented with 0.1% of dimethyl sulfoxide (DMSO) was used as a control.

### 2.2. Extraction of Cranberry Pomace

Frozen cranberries were kindly provided by the local company Fudo (Kaunas, Lithuania). The berries were thawed and the juice immediately pressed in a Philips HR1880/01 juicer. The pomace was dried at 35 °C in a hot air dryer until a final moisture content of 5.83%. The dry pomace was milled and extracted by supercritical carbon dioxide in a 10 L extractor (Applied Separations, Allentown, PA, USA) to remove lipophilic substances [41]. Pressurized liquid extraction (PLE) of defatted pomace powder was mixed with diatomaceous earth, placed in 50 mL extraction cells, which were fixed with cellulose filters at the top and bottom, and extracted with water in an accelerated solvent extraction apparatus ASE350 (Dionex, Sunnyvale, CA, USA) at a constant 10.3 MPa pressure [41]. The yield of the water extracts was 6.50%. Applied PLE parameters for water extraction were previously reported [42]. After extraction, the extract was freeze-dried. Cranberry pomace extracts were dissolved in DMSO (40 mg/mL) and kept at −20 °C in the dark until further investigation.

### 2.3. Phytochemical Characterization of Cranberry Pomace Extracts

The main phytochemicals in the extract were quantified using Waters ACQUITY ultra performance liquid chromatography system (Waters Corp., Milford, MA, USA), equipped with hybrid Brucker Daltonics (Bremen, Germany) time-of-flight/quadrupole mass detector (UPLC-Q/TOF) and an Acquity BEH, C18 column (2.1 × 100 mm, particle size 1.7 μm) (Waters Corp., Dublin, Ireland). Detailed analytical data may be found elsewhere [41].

### 2.4. Cell Viability Assay

Mitochondrial activity was measured using the colorimetric assay tetrazolium salt MTT (3-[4,5-dimethylthiazol-2-yl]-2,5-diphenyltetrazolium bromide) in A549 or Huh7.5 cells. One hundred microliters of two-fold dilutions of cranberry pomace extract ranging from 25 to 2000 µg/mL were inoculated on A549 or Huh7.5 cells cultured in a 96-well plate. After 48 h of cell treatment, the supernatant was removed; the cells were washed twice with PBS; and then 120 µL of MTT solution was added. The plate was further incubated for 2 h at 37 °C under 5% CO_2_ atmosphere. The supernatant was discarded and the formazan crystals were solubilized by adding 50 µL of DMSO (Sigma-Aldrich, Saint-Quentin-Fallavier, France). Absorbance was measured at 570 nm with a reference wavelength at 690 nm. The CC_50_ was determined using a nonlinear regression on the GraphPad Prism software (version 9.00, La Jolla, CA, USA).

### 2.5. Flow Cytometry Assay

Cells were trypsinated and fixed with 3.7% PFA in PBS for 20 min. A solution of Triton X-100 (0.15%) in PBS was used to permeabilize the cells after which they were stained by incubation with the mouse anti-pan flavivirus envelope E protein mAb 4G2-conjugated to Alexa Fluor 488-labeled (RD-Biotech, Besançon, France) (1:1000 dilution) for 1h at RT. The cells were then processed by flow cytometric analysis with Cytoflex (Beckman Coulter, Brea, CA, USA). The data were analyzed using CytExpert software (Brea, CA, USA).

### 2.6. Virus Inactivation Assay

The direct effect on ZIKV particles was evaluated by incubating ZIKV^GFP^-free particles (2 × 10^5^ PFU) with CP extract for 2 h at 37 °C. As a control, ZIKV^GFP^ particles were incubated with a culture medium. A 50-fold dilution with MEM containing 5% FBS was assessed to dilute the extract at a non-therapeutic concentration thereby preventing significant interactions with cellular receptors. The final mixtures were inoculated on A549 cells that had already been seeded in 24-well plates. The cells were collected, fixed and handle counted by cytometry assay 24 h post-incubation.

### 2.7. Virus Binding Assay

Cells, pre-chilled at 4 °C, were incubated with ZIKV^GFP^ at a multiplicity of infection (MOI) of 2 in the presence or absence of sample for 1 h on ice. The supernatant was then discarded and the cells were washed twice with PBS. A fresh medium was then added, and the cells were further incubated for 24 h at 37 °C before being subjected to flow cytometry.

### 2.8. Plaque Forming Assay

Vero cells were seeded in 24-well culture plates at a density of 7 × 10^4^ cells per well and incubated at 37 °C for 24 h prior to infection [21]. Thirty microliters of 10-fold dilution of supernatants were inoculated on the Vero cells. Two hours post-infection, 0.2 mL of 0.8% carboxymethylcellulose sodium salt solution (Sigma-Aldrich, Saint-Quentin-Fallavier, France) prepared in MEM 5% FBS was added, and the plates were then incubated for 4 (ZIKV) or 5 days (DENV) at 37 °C. To reveal the viral titer, the supernatant was discarded gently and the cells were washed twice with PBS, fixed with PFA 3.7% and dyed with a solution of 0.5% crystal violet (Sigma-Aldrich, Saint-Quentin-Fallavier, France). The plaques were counted and represented as plaque-forming unit per mL (PFU/mL).

### 2.9. Immunofluorescence Assay

Infected cells seeded on coverslips were fixed with 3.7% PFA for 10 min and permeabilized with Triton X-100 (0.15%) in PBS for 4 min. Adhered cells were then labeled for 1 h at RT with the mouse anti-pan flavivirus envelope E protein mAb 4G2 (1:1000 dilution), and the nucleus was stained with DAPI [21]. The glass coverslips were then deposed inverted in the mounting medium Vectashield (VectorLabs). A fluorescent signal was observed using a Nikon Eclipse E2000-Umicroscope and images were taken using the Hamamatsu ORCA-ER camera and NIS-Element AR (Nikon) imaging software.

### 2.10. Zebrafish Maintenance, Intraperitoneal Injection and Behavior Monitoring

Adult wild-type zebrafish (Danio rerio; 1 year old, mixed sex) were kept in standard conditions of temperature (28 °C), photoperiod (14/10 h light/dark), and conductivity (400 μS). The fish were supplied with commercial food from Planktovie (Gemma Micro ZF 300) 3 times per day. For intraperitoneal extract administration, fish were anesthetized with 0.02% tricaine (MS-222; REF: A5040, Sigma-Aldrich) before being administered with either (5% MEM) or cranberry pomace extract (400 µg/g body weight). The fish were returned immediately to the water and closely monitored for any obvious signs of stress (e.g., changes in locomotor behavior, feeding). One day after injection, the fish were analyzed for locomotion with ZebraCube equipment (Viewpoint). Individual control fish and treated fish were placed in separate tanks containing 800 mL of water within the ZebraCube. After a 5-min adaptation period, locomotion was monitored for 10 min to determine its state: inactivity (<4 mm/s), low activity (4–8 mm/s), or high activity (>8 mm/s). A total of 7 control versus 7 treated-fish were closely analyzed at 1 dpi for the locomotor activity. In addition, fish survival was monitored until day 5. At the end of the experimental procedure, fish were sacrificed with an overdose of tricaine.

### 2.11. Statistical Analysis

A one-way ANOVA test was used to compare the different concentrations. Values were expressed as the mean ±SD of a minimum of three independent experiments. All statistical analyses were carried out using GraphPad Prism software (version 9.0; La Jolla, CA, USA). Significance levels are shown in the figure as follows: * *p* < 0.05; ** *p* < 0.01; *** *p* < 0.001, **** *p* < 0.0001, n.s. = not significant.

## 3. Results

### 3.1. Cranberry Pomace Extract Doses Did Not Exhibit Toxicity in Adult Zebrafish

We recently characterized the phytochemical composition of an agrifood byproduct extract, cranberry pomace (CP), recovered after cranberry juice extraction [41]. In total, 8 phytochemicals were identified, 3 of which were quantified using analytical standards [41]. Quinic acid was a major compound having a concentration of 869 ± 3 mg/100 g. The concentrations of anthocyanins peonidin-3-galactoside and peonidin-3-arabinoside were 0.96 ± 0.02 and 0.86 ± 0.01 mg/100 g, respectively [41].

**Figure 1:**
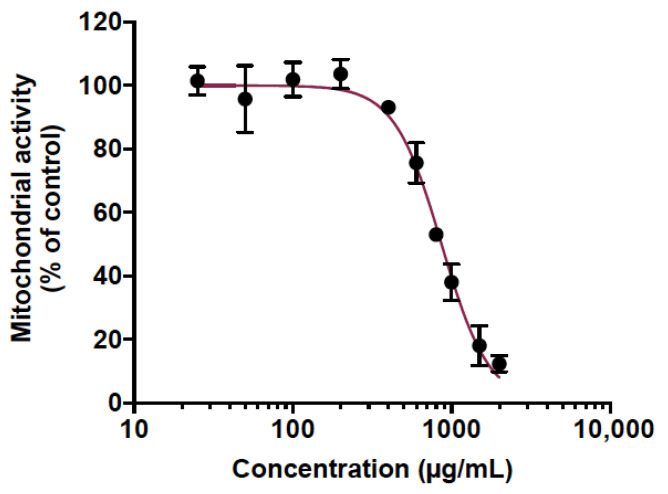
Determination of the maximal non-toxic concentration (MNTC) of cranberry pomace extract on human A549 cell line. A549 cells were grown in the presence of various concentrations of CP extract (25 to 2000 µg/mL) for 48 h. The metabolic activity was assessed by MTT assay. Results presented are means ±SD of three independent experiments and are expressed as relative values to the vehicle control.

Animal testing was used to predict the toxicity of CP extract. This is mounting evidence that zebrafish is a relevant in vivo model to predict human toxicity in pharmaceutical development [43,44]. Indeed, a large genomic similarity with humans (>70%) as well as evolutionarily preserved physiological processes with mammals make the zebrafish a very interesting and popular model for toxicity studies [43,44]. Here, the potentially acute toxicity of CP extract was determined in adult zebrafish. For this purpose, we initially determined the maximum non-toxic concentration (MNTC) in vitro on human epithelial cell lines A549 using an MTT assay. Cells were treated with various concentrations of CP extract ranging from 25 to 2000 µg/mL (Figure 1). The results displayed a concentration-dependent toxicity where the cytotoxic concentration to inhibit 50% of mitochondrial activity (CC_50_) was found to be 865.1 µg/mL (Figure 1). The maximum non-toxic MNTC concentration (>90% viability) was determined to be 400 µg/mL (Figure 1). Therefore, this concentration was chosen to test the toxicity of the PC extract on adult zebrafish.

Consequently, to determine if this dose could be toxic in vivo, an intraperitoneal injection of CP extract was performed in adult zebrafish using MNTC of 400 µg/g of body weight. The experiment was carried out for 5 days, and fish survival was monitored several times a day throughout. We did not detect any sign of suffering, stress or abnormal locomotor or feeding behavior (Table 1). In addition, the results showed that from injection to day 5, CP extract-injected fish displayed 100% survival, similar to the vehicle control (Table 1). Thus, CP extract at concentration of 400 µg/g of body weight does not exhibit obvious toxicity in vivo.

### 3.2. Cranberry Pomace Extract Exerts Antiviral Activity against the Epidemic ZIKV Strain

**Figure 2:**
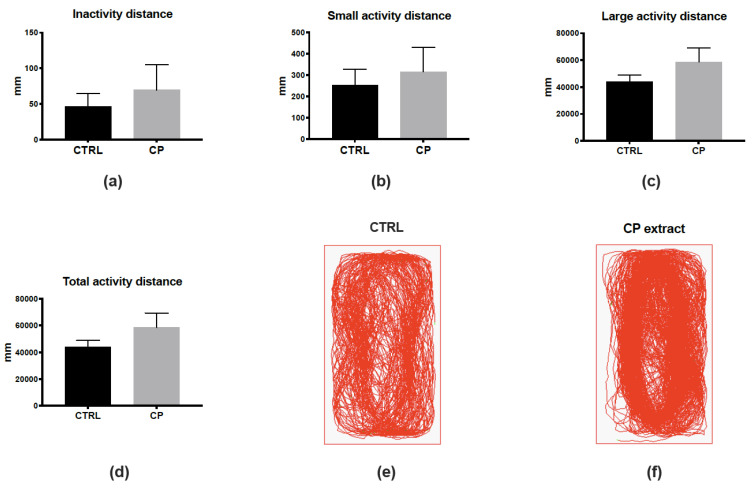
Cranberry pomace extract has no impact on finely tuned locomotor activity one day after injection. (**a**–**c**) Distance traveled in the “inactive” (<4 mm/s, (**a**)), low activity (4–8 mm/s, (**b**)), and high activity (>8 mm/s, **c**) states during the 10-min recording period. (**d**) Total distance traveled by the fish during the recording period. Note that no significant differences were observed between groups. (**e**,**f**) Examples of paths traveled by control and CP-injected fish. *n* = 7 fish/group; data represent means ± SEM.

To reinforce the fact that CP extract could be non-toxic in vivo, as the MNTC determined in vitro, we decided to analyze the behavior of control and CP-injected fish (Figure 2). To this aim, we monitored the locomotion of fish one day after the injection, defining three different states of activity: inactivity (<4 mm/s), low activity (4–8 mm/s), or high activity (>8 mm/s). The monitoring and analysis of fish locomotion did not show significant differences in these three states or in the total distance traveled (Figure 2a–d). The general pattern of locomotion was also highly similar between control and CP-injected fish (Figure 2e,f). Together, these data suggest the absence of any striking effects of the injection of CP extract considering toxicity and behavior.

**Figure 3:**
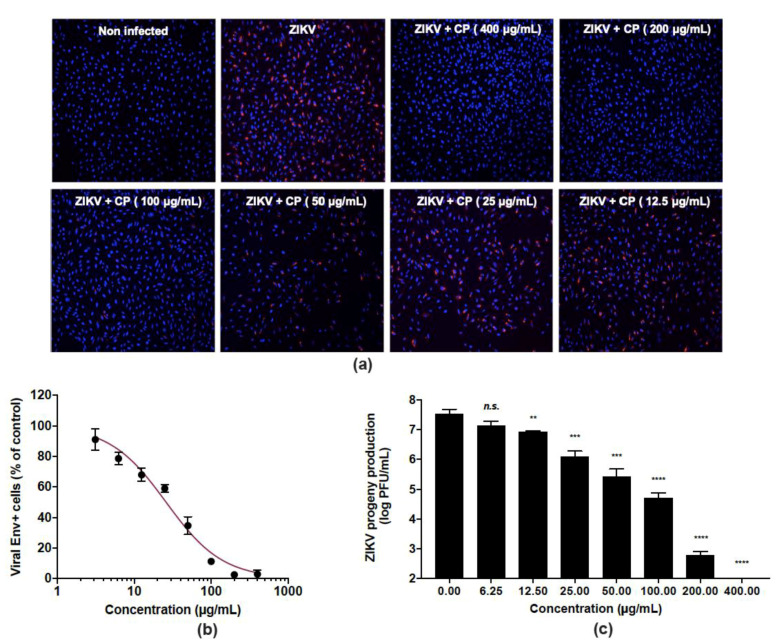
Cranberry pomace extract exhibits dose-dependent antiviral effect against the epidemic strain of ZIKV. A549 cells were challenged with the epidemic strain of ZIKV (PF-25013-18) at an MI of 2 and simultaneously treated with various non-cytotoxic concentrations of CP extract (0–400 µg/mL). (**a**) Immunofluorescence analysis of viral protein expression in A549-ZIKV-PF13-infected cells. The ZIKV E (red) and nuclei (blue) were visualized by fluorescence microscopy. Images are representative of three independent experiments. (**b**) Quantification of the number of cells positive for the viral protein E expression in A549-ZIKV-infected cells by immunofluorescence. (**c**) ZIKV growth was assessed by a plaque formation assay. Results from a representative experiment (*n* = 3 repeats) are shown. Data are means ± SD of three separate experiments. One-way ANOVA and Dunnett’s test were used for statistical analysis (** *p* < 0.01; *** *p* < 0.001; **** *p* < 0.0001; n.s. = not significant).

The ability of CP extract to inhibit ZIKV infection was evaluated in human epithelial A549 cells infected by the clinical isolate of the epidemic strain PF-25013-18 of ZIKV [39]. The CP extract showed no cytotoxicity in A549 cells at a concentration up to 400 µg/mL (Figure 1). A549 cells were infected for 24 h with ZIKV at a multiplicity of infection (MOI) of 2 in the presence of non-toxic concentrations in vivo (6.25–400 µg/mL) of CP extract (Figure 3). An immunofluorescence assay using anti-flavivirus E mAb 4G2, showed that the CP extract had a dose-dependent anti-ZIKV activity (Figure 3a). At 400 µg/mL, CP extracts completely protected the A549 cell monolayer from ZIKV infection (Figure 3a), and 100 µg/mL of CP extract was sufficient to reduce the number of ZIKV infected cells by more than 90% (Figure 3b). The concentration that inhibited 50% of ZIKV infection (IC_50_) was estimated to be 26.0 µg/mL using nonlinear regression after construction of a concentration-activity sigmoid curve. (Figure 3b). Thus, the selectivity index (SI), which measures the benefit/risk ratio, was calculated as CC_50_/IC_50_ = 33.2. Viral growth was strongly inhibited in a dose-dependent way when non-toxic concentrations of CP extract were present. (Figure 3c). At MNTC, the CP extract had ability to suppress viral progeny production (Figure 3c). At a concentration of 100 µg/mL, the extract further reduced virus progeny production by up to 2 logs. (Figure 3c).

Taken together, these data suggest that the CP extract is a potent antiviral against ZIKV at concentrations devoid of toxicity in vivo.

### 3.3. Cranberry Pomace Extract Inhibits ZIKV Binding to the Host Cell

**Figure 4:**
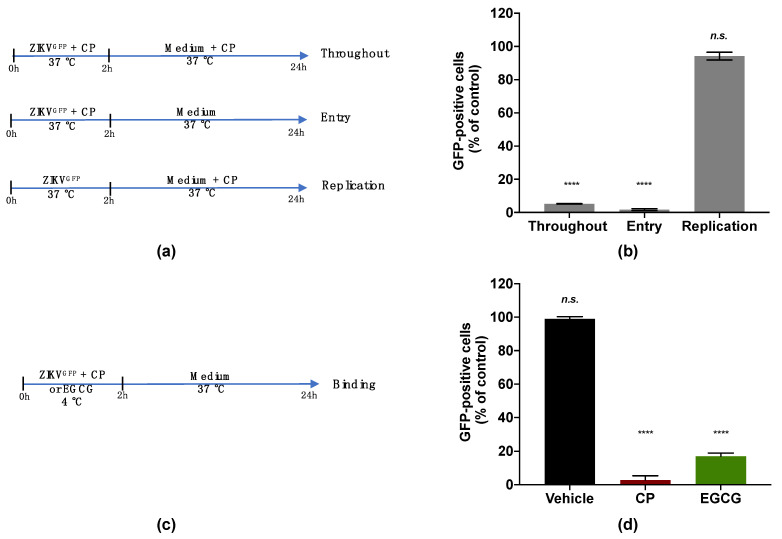
Cranberry pomace extract inhibits the attachment of ZIKV to the surface of A549 cells. (**a**) Schematic illustration of drug addition time assays applied to describe the antiviral activity of CP extract (100 µg/mL). A549 cells were infected and treated with CP extract or vehicle throughout infection (Throughout), simultaneously with virus entry (Entry), after virus challenge (Replication) with appropriate time-wash steps and incubation periods. (**b**) Flow cytometric analysis of infected cells under the different experimental conditions shown in (**a**). (**c**) Schematic illustration of the binding assay. Pre-chilled ZIKV^GFP^ was mixed with CP extract (100 µg/mL) and left to bind to the A549 cell monolayer at 4 °C for 1 h, then moved to 37 °C. EGCG (100 µM) was used as a positive control. (**d**) Flow cytometric analysis of cells infected with ZIKV^GFP^-A549 in the binding assay shown in (**c**). The results are means ±SD of three independent experiments and are expressed as relative values to vehicle infected cells. One-way ANOVA and Dunnett’s test were used for statistical analysis (**** *p* < 0.0001; n.s. = not significant).

To investigate the viral target of the CP extract, a ZIKV mutant expressing the autofluorescence reporter protein GFP (ZIKV^GFP^) was used [40]. The expression level of GFP related to the efficacy of ZIKV infection in the host cell. The antiviral effect of the CP extract was first validated against ZIKV^GFP^ when a 90% reduction of GFP-positive A549 cells was observed at 100 µg/mL (Figure S1). A time-of-drug addition assay was carried out to identify which stage of the ZIKV infection cycle can be affected by the CP extract (Figure 4a). The number of GFP-expressing cells was lower by 90% compared with the control when 100 µg/mL of the extract was added during the whole experiment. (Figure 4b). Similar results were obtained when the extract was added concomitantly with virus input to cover the entry step and yielded a 90% reduction in GFP-positive cells (Figure 4b). However, no antiviral activity was observed when the CP extract was added after 2 h of viral challenge (Figure 4b). These results showed that the CP extract effect was mediated by inhibition of the early stage of the ZIKV infectious cycle. Indeed, the inhibition of viral infection was not correlated with the replication step but rather with the inability of ZIKV to initiate an infectious cycle in the host cell in the presence of the CP extract.

We then evaluated the capacity of the CP extract to interfere with ZIKV attachment to the host cell surface (Figure 4c). For this, a binding assay was carried out at 4 °C to allow virus attachment but not internalization (Figure 4c). Briefly, pre-chilled ZIKV^GFP^ was mixed with the extract (100 µg/mL) and left to bind to the A549 cell monolayer at 4 °C for 1 h then moved to 37 °C (Figure 4c). Epigallocatechin gallate (EGCG) at 100 µM, a polyphenol from green tea known to block ZIKV attachment [22,29], was used as a positive control. Our data showed that the percentage of A549-infected cells was significantly reduced in the presence of the CP extract as well as EGCG (Figure 4d). This reduction in infected cells compared to control suggested that the inability of ZIKV to initiate an infectious cycle in the presence of the extract was associated with the failure of virus attachment. Taken together, these results showed that the CP extract prevented ZIKV entry into the host cell by inhibiting the viral binding step.

**Figure 5:**
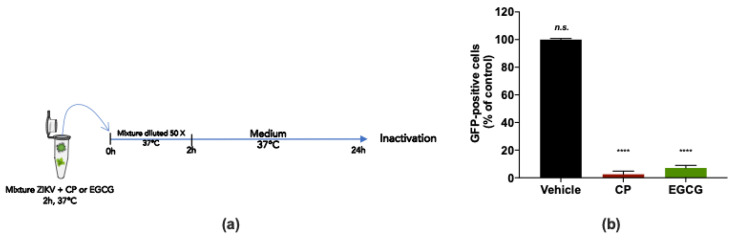
Cranberry pomace extract prevents virus entry by acting directly on ZIKV particles. (**a**) Schematic illustration of ZIKV inactivation assay performed to characterize the virucidal activity of the CP extract. ZIKV^GFP^ was untreated (vehicle) or treated with the CP extract (100 µg/mL) for 2 h at 37 °C and subsequently diluted 50 times before infection of A549 cells. EGCG (100 µM) was used as a positive control. (**b**) Flow cytometric analysis under the experimental inactivation conditions shown in (**a**). The results are means ±SD of three independently performed experiments and are expressed as relative values to untreated infected cells. One-way ANOVA and Dunnett’s test were used for statistical analysis (**** *p* < 0.0001; n.s. = not significant).

To examine whether the CP extract may affect ZIKV infectivity, a virus inactivation assay was conducted (Figure 5a). For this purpose, a 2 × 10^5^ PFU dose of ZIKV^GFP^ was incubated with 100 µg/mL CP extract for 2 h at 37 °C and subsequently diluted 50 times before infection of A549 cells (Figure 5a). EGCG (100 µM), known for its virucidal activity [22,29], was used as a positive control (Figure 5a). By FACS analysis, we observed that the CP extract and EGCG reduced the number of GFP-positive A549 cells by at least 95% compared to mock-treated infected cells (Figure 5b). Taken together, the findings suggest that the CP extract has a virucidal effect on ZIKV, thereby blocking viral entry into the host cell.

### 3.4. Cranberry Pomace Extract Acts on Dengue Virus

**Figure 6:**
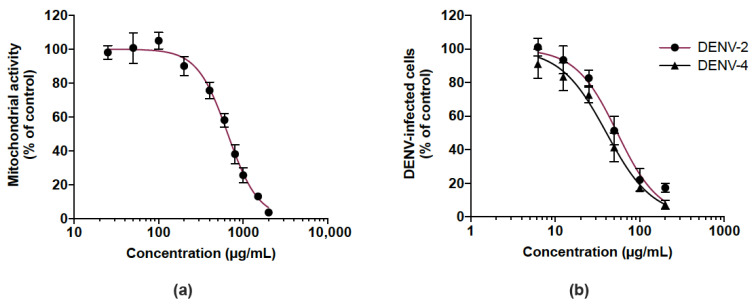
Cranberry pomace extract exhibits antiviral effect against two DENV serotypes. (**a**) Mitochondrial activity of Huh7.5 cells incubated with different concentrations of CP extract. Cells were grown in the presence of increasing concentrations of CP extract (25 to 2000 µg/mL) for 48 h. The mitochondrial activity of the cells was assessed by MTT assay. (**b**) Huh7.5 cells were infected for 48 h with DENV-2 or DENV-4 (MI 0.5) in the presence of various non-cytotoxic concentrations of CP extract (6.25–200 µg/mL). Flow cytometric analysis was performed using the anti-flavivirus E mAb 4G2. Data represent means ±SD of three independent experiments.

We then examined whether the CP extract exerted antiviral activity against DENV, another medically relevant flavivirus, so we investigated its antiviral potential against DENV-2 and DENV-4. Given that human hepatoma Huh7.5 cells support DENV replication, Huh7.5 cells were first assessed for their sensitivity to the extract using a MTT assay. As shown in Figure 6a, the Huh7.5 cell line had an estimated CC_50_ of 656.1 µg/mL. DENV-infected Huh7.5 cells were treated with different non-toxic concentrations (up to 200 µg/mL) of the CP extract over 48 h. Cells infected by DENV-2 and DENV-4 were detected by a FACS assay using anti-E mAb 4G2. There was a similar dose-dependent reduction of DENV-infected Huh7.5 cells in the presence of the CP extract regardless of the DENV serotype tested (Figure 6b). The estimated IC_50_ of DENV-2 and DENV-4 are 54.2 and 40.3 µg/mL, respectively. These results showed that the CP extract exerted an antiviral effect against different serotypes of DENV as already observed with ZIKV.

## 4. Concluding Remarks

The use of plant byproducts as antiviral nutraceuticals represents a cost-effective and environmentally friendly approach [20,26,45]. Due to their abundance in food products and their potential beneficial pharmacological and nutritional effects, polyphenols are of considerable interest as a drug and dietary supplement. In this study, we aimed to valorize a polyphenol-rich agrifood byproduct, cranberry pomace extract [41], for its nutraceutical antiviral potential against emerging arboviruses such as dengue and zika. Antivirals are described as virostatic or virucidal if they inactivate or destroy viral particles, respectively, resulting in a loss of infectivity [20]. This mechanism of action has been described for various natural compounds such as EGCG, delphinidin, curcumin, geraniin and berberine against ZIKV and DENV [22,23,31,46,47]. Numerous studies have highlighted the capacity of medicinal plants extracts to neutralize ZIKV or DENV infectivity [19,21,22,23,24,25,30,31,48,49]. Here, using a panel of virological assays, we demonstrated that cranberry pomace extract, as well as EGCG, is capable of neutralizing the infectivity of the Asian epidemic strain of ZIKV in zebrafish at non-toxic doses. We also demonstrated that the CP extract prevents the infection of human cells by two clinical isolates of dengue, serotypes 2 and 4. A time-of-drug addition assay and binding and inactivation assays suggest that inhibition of ZIKV was related to the inability of viral particles to interact with cellular receptors. The phytochemical composition of the CP extract was characterized recently using UPLC-QTOF-MS highlighting the presence of quinic acid as the major compound [41]. Quinic acid derivatives have already been shown to be active against DENV [50], where the study conducted by Zanello et al. Ref. [50] demonstrated that the amides of quinic acid derivatives inhibit the replication step of dengue virus in Huh7.5 cells while not exhibiting a direct effect on the viral particles. However, our virological assays showed that the CP extract did not act at the replication step; rather, it acted directly on the viral particles to prevent their attachment to the host cell surface. Based on this mechanism of action, it seems reasonable to conclude that quinic acid was not involved in the antiviral effect of CP extract. The phytochemical composition of the CP extract also revealed the presence of quercetin hexoside and anthocyanins such as peonidin-3-galactoside and peonidin-arabinoside [41]. We recently showed that a quercetin-hexoside molecule (isoquercetrin) prevented the internalization of ZIKV in A549 cells. The involvement of quercetin-hexoside, present in the CP extract, in inhibiting viral entry remains to be investigated once the virucidal compounds are separated and isolated. On the other hand, it has been demonstrated that an anthocyanin named delphinidin exerts a virucidal effect against various flaviviruses among which are ZIKV and DENV. It would be interesting to verify that the anthocyanins detected in the CP extract, such as peonidin-3-galactoside and peonidin-arabinoside, are potentially involved in the virucidal effect of the CP extract.

The virucidal and virostatic effects of polyphenols such as EGCG and anthocyanin as delphinidin remain to be better understood. EGCG causes a loss of viral particle integrity, which limits interactions with membrane receptors at the surface of the host cell [22]. Delphinidin targets cell surface proteins involved in the viral attachment process preventing virus attachment [51]. In this way, molecular docking studies demonstrated a flavonoid binding pocket on the surface of the viral envelope protein E [52]. Although further studies are needed, it seems reasonable to assume a similar mechanism of action for the active compounds in the CP extract. Thus, it would be very interesting to identify the active phytochemical compounds that act as antiviral agents or if there is a synergistic effect of several compounds that provides antiviral activity.

In conclusion, we demonstrated for the first time that cranberry pomace extract exerts an antiviral effect against ZIKV and DENV at a concentration that had no effect on zebrafish, a well-recognized small animal model ideal for drug toxicity assessment [43,44]. Therefore, our study raises the opportunity to use the agrifood byproduct derived from berry extract as a potential source of safe and effective antivirals against medically important mosquito-borne viruses.

## Figures and Tables

**Table 1:** Survival of fish injected with cranberry pomace extract from 1 day to 5 days post injection (dpi).

Number of Fish Alive							
	Number of Injected Fish	1 dpi	2 dpi	3 dpi	4 dpi	5 dpi	Survival Rate at 5 dpi (%)
Vehicle control	15	15	15	15	15	15	100
CP extract	18	18	18	18	18	18	100

## Data Availability

Not applicable.

## Supplementary Materials

The following supporting information can be downloaded at: https://www.mdpi.com/article/10.3390/v14051101/s1, Figure S1: Cranberry pomace extract exhibits a dose-dependent antiviral activity against ZIKV^GFP^.
