# Supplementary material for: Cranberry Pomace Extract Exerts Antiviral Activity against Zika and Dengue Virus at Safe Doses for Adult Zebrafish
Source: Viruses. 2022 May 20;14(5):1101. doi: 10.3390/v14051101 (PMC9147401; doi:10.3390/v14051101)
Supplement: Supplementary file 1 [file viruses-14-01101-s001.zip › viruses-1683372-supplementary.pdf]

viruses

MDPI

Article

Cranberry Pomace Extract Exerts Antiviral Activity against Zika and Dengue Virus at Safe Doses for Adult Zebrafish

Laura Tamkutė [1,2,†], Juliano G. Haddad [1,†], Nicolas Diotel [3], Philippe Desprès [1], Petras Rimantas Venskutonis [2] and Chaker El Kalamouni [1,*]

[1] Université De La Réunion, INSERM U1187, CNRS UMR 9192, IRD UMR 249, Unité Mixte Processus Infectieux En Milieu Insulaire Tropical, Plateforme Technologique CYROI, 94791 Sainte Clotilde, France; laura.tamkute@ktu.lt (L.T.); juliano.haddad@univ-reunion.fr (J.G.H.); philippe.despres@univ-reunion.fr (P.D.)

[2] Department of Food Science and Technology, Kaunas University of Technology, Radvilenų pl. 19, LT-50254 Kaunas, Lithuania; rimas.venskutonis@ktu.lt

[3] Université de La Réunion, INSERM, UMR 1188 Diabète Athérothrombose Thérapies Réunion Océan Indien (DéTROI), 97490 Saint-Denis de La Réunion, France; nicolas.diotel@univ-reunion.fr

* Correspondence: chaker.el-kalamouni@univ-reunion.fr

† These authors contributed equally to this work.

Citation: Tamkutė, L.; Haddad, J.G.; Diotel, N.; Desprès, P.; Venskutonis, P.R.; El Kalamouni, C. Cranberry Pomace Extract Exerts Antiviral Activity against Zika and Dengue Virus at Safe Doses for Adult Zebrafish. *Viruses* **2022**, *14*, x. https://doi.org/10.3390/xxxxx

Academic Editor(s): Leen Delang

Received: 31 March 2022
Accepted: 20 May 2022
Published: 20 May 2022

Publisher's Note: MDPI stays neutral with regard to jurisdictional claims in published maps and institutional affiliations.

Copyright: © 2022 by the authors. Submitted for possible open access publication under the terms and conditions of the Creative Commons Attribution (CC BY) license (https://creativecommons.org/licenses/by/4.0/).

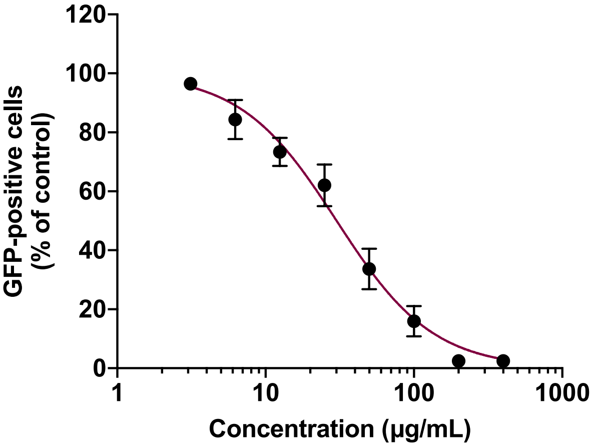

Figure S1. Cranberry pomace extract exhibits a dose-dependent antiviral activity against ZIKV^{GFP}. A549 cells were infected with the recombinant molecular clone ZIKV^{GFP} at MI of 1 and continuously treated with different non cytotoxic concentrations of CP extract (0 – 400 µg/mL). Flow cytometric analysis of GFP fluorescence was performed 24 hours post infection. The results shown are means ± SD of four independent experiments and are expressed as relative value compared to untreated infected cells.
